# Supplementary material for: Chromothripsis during telomere crisis is independent of NHEJ, and consistent with a replicative origin
Source: Genome Res. 2019 May;29(5):737–49. doi: 10.1101/gr.240705.118 (PMC6499312; doi:10.1101/gr.240705.118)
Supplement: Supplemental Material [file supp_gr.240705.118_Supplemental_file_1.zip › contigs/annotated_contigs/DB68/contig.2.DB68_length_432_mean_cov_3.81944444444.docx]

**DB68_length_432_mean_cov_3.81944444444**

```
ATGGTTGAGTCTGTGAGGTTTTCATTCTTTC|ATTCATCCTGTTT|ATAATATTCTCCAGGCTGAATGAAGACAGCTGTATAAACTTAA
 >chr7:109084449-109084480 + E=4e-07 p=3e-03  >chr7:109084848-109085236 - E=1e-222
CTTTATACTGAAAGTTATGTGAACCTTGTAAACTATTTTTGTTTTAAAATATTGGAGTTATATGAAATATGTGGAAAAAATAAAAATGA

TGAATATAAGAAGCATTTCAATTTTGTCTATCAAACATACAAATGAACATATAATACAAAATTATTTTTGCATAAGCAGAATAATATTT

CCATTAGTTTGCACAATACTACTACACACAATTTATTCAGGGAAACCAGATGAAGTAGTAAAAGTGAAGCTGTCTTGTGGATATTTCAT

TTGTTCTGTGGTAAACAAAATTGTTTTGAGGAAACACCATATATATTAACTACCATCCTGTTTCATAAACAGTTCAAA
```
